# Renal effects and safety between Asian and non‐Asian chronic kidney disease and type 2 diabetes treated with nonsteroidal mineralocorticoid antagonists

**DOI:** 10.1111/1753-0407.13566

**Published:** 2024-05-16

**Authors:** Xiaoming Xu, Jing Feng, Yuying Cui, Pingjiang Li, Jianjun Dong, Lin Liao

**Affiliations:** ^1^ Department of Endocrinology and Metabology, Shandong Provincial Qianfoshan Hospital Shandong University Jinan China; ^2^ Department of Endocrinology and Metabology The First Affiliated Hospital of Shandong First Medical University & Shandong Provincial Qianfoshan Hospital Jinan China; ^3^ First Clinical Medical College Shandong University of Traditional Chinese Medicine Jinan China; ^4^ Shandong First Medical University and Shandong Academy of Medical Sciences Jinan China; ^5^ Department of Endocrinology Qilu Hospital of Shandong University Jinan China

**Keywords:** Asians, chronic kidney disease, non‐Asians, nonsteroidal mineralocorticoid receptor antagonists, type 2 diabetes mellitus

## Abstract

**Background:**

Asians bear a heavier burden of chronic kidney disease (CKD), a common comorbidity of type 2 diabetes mellitus (T2DM), than non‐Asians. Nonsteroidal mineralocorticoid receptor antagonists (MRAs) have garnered attention for their potential advantages in renal outcomes. Nevertheless, the impact on diverse ethnic groups remains unknown.

**Methods:**

The PubMed, Embase, Cochrane Library, China National Knowledge Infrastructure (CNKI), Wanfang database, and clinical trial registries were searched through August 2023 with the following keywords: nonsteroidal MRAs (finerenone, apararenone, esaxerenone, AZD9977, KBP‐5074), CKD, T2DM, and randomized controlled trial (RCT). A random effects model was used to calculate overall effect sizes.

**Results:**

Seven RCTs with 14 997 participants were enrolled. Nonsteroidal MRAs reduced urinary albumin to creatinine ratio (UACR) significantly more in Asians than non‐Asians: (weighted mean difference [WMD], −0.59, 95% CI, −0.73 to −0.45, *p* < .01) vs (WMD, −0.29, 95% CI, −0.32 to −0.27, *p* < .01), respectively. The average decline of estimated glomerular filtration rate (eGFR) was similar in Asians and non‐Asians (*p* > .05). Regarding systolic blood pressure (SBP), nonsteroidal MRAs had a better antihypertension performance in Asians (WMD, −5.12, 95% CI, −5.84 to −4.41, *p* < .01) compared to non‐Asians (WMD, −3.64, 95% CI, −4.38 to −2.89, *p* < .01). A higher incidence of hyperkalemia and eGFR decrease ≥30% was found in Asians than non‐Asians (*p* < .01).

**Conclusions:**

Nonsteroidal MRAs exhibited significant renal benefits by decreasing UACR and lowering SBP in Asian than that of non‐Asian patients with CKD and T2DM, without increase of adverse events except hyperkalemia and eGFR decrease ≥30%.

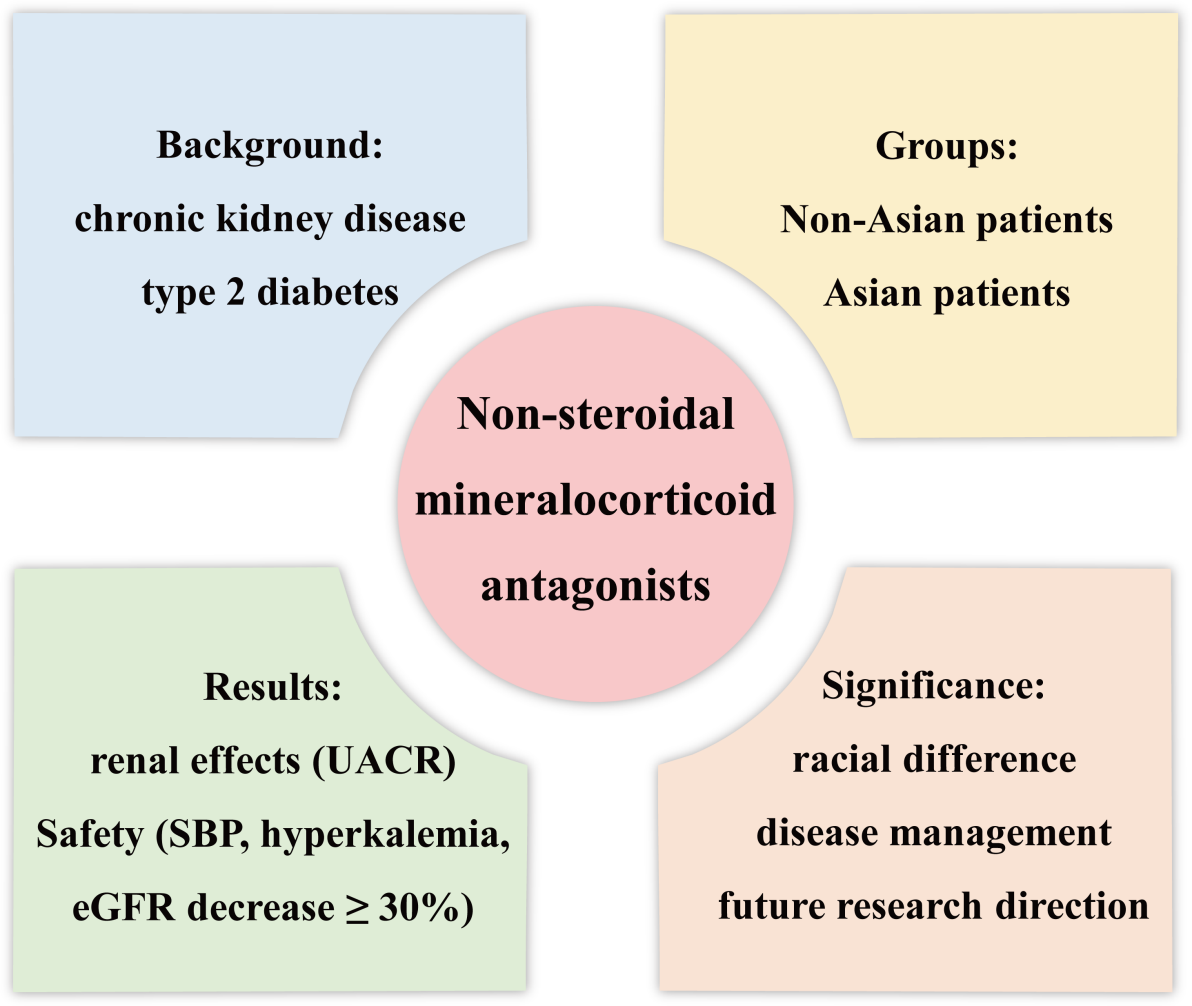

## Graphical abstract

Highlights

Our study indicated that nonsteroidal mineralocorticoid receptor antagonists decreased urinary albumin to creatinine ratio and systolic blood pressure significantly greater in Asian chronic kidney disease and type 2 diabetes mellitus patients than non‐Asian patients. The average decline in estimated glomerular filtration rate (eGFR) did not show racial preference. However, hyperkalemia and eGFR decrease ≥ 30% occurred more frequently in Asians, which was acceptable without renal failure or death.

## 1 INTRODUCTION

Diabetes remains a substantial public health issue, relevant to the changes in human behavior and lifestyle.^1^ The most common is type 2 diabetes mellitus (T2DM), with estimated prevalence of 10%–25% in Asian countries and 5%–15% in non‐Asian countries.^2^ Among the approximately 463 million adults with diabetes, nearly half live in China, followed by India.^3^ China also harbors the largest population affected by chronic kidney disease (CKD),^4^ despite a prevalence rate of 10.08% lower than global average 13.4%.^5^ These data highlight the disproportionate global burden of diabetes and CKD, which is more pronounced in Asian than in non‐Asian regions. With the aging of the population and the increasing incidence of diabetes, the prevalence of albuminuria and reduced eGFR is still on the rise.^6^

Evidence suggests that overactivation of the mineralocorticoid receptor (MR) leads to inflammation and fibrosis that can drive CKD and cardiovascular disease progression.^7^ Nonsteroidal MR antagonists (MRAs; including finerenone, apararenone, esaxerenone, AZD9977, and KBP‐5074) with higher selectivity than steroidal MRAs are currently in development.^8^, ^9^ The FIDELITY pooled analysis, combined two phase III trials FIDELIO‐DKD (Efficacy and Safety of Finerenone in Subjects With Type 2 Diabetes Mellitus and Diabetic Kidney Disease; NCT02540993) and FIGARO‐DKD (Efficacy and Safety of Finerenone in Subjects With Type 2 Diabetes Mellitus and the Clinical Diagnosis of Diabetic Kidney Disease; NCT02545049), provided valuable insights into the cardiovascular and renal protective effects of finerenone in patients with CKD and T2DM.^10^, ^11^, ^12^

There are many articles on the efficacy and safety of nonsteroidal MRAs,^13^, ^14^, ^15^, ^16^ but there is a lack of systematic comparison in different subgroups. Thus, we performed this meta‐analysis to evaluate the different renal outcomes and side effects between Asian and non‐Asian CKD and T2DM patients when treated with nonsteroidal MRAs.

## 2 METHODS

### 2.1 Data sources and search strategy

This systematic review and meta‐analysis was performed following the Preferred Reporting Items for Systematic Reviews and Meta‐Analyses (PRISMA) statement.^17^ The protocol is available from the International Database of Prospectively Registered Systematic Reviews (PROSPERO: CRD42023445719).

A systematic literature search of PubMed, Embase, Cochrane Library, China National Knowledge Infrastructure (CNKI), Wanfang database, and clinical trial register centers (http://www.clinicaltrials.gov) was performed for relevant clinical trials published until 28 August 2023, without language restrictions. The following Medical Subject Headings (MeSH) terms and text words were used in different search combinations: nonsteroidal mineralocorticoid receptor antagonists (finerenone, apararenone, esaxerenone, AZD9977, KBP‐5074), chronic renal insufficiency, type 2 diabetes mellitus, and randomized controlled trials.

### 2.2 Study selection and criteria

Eligible studies met the following requirements: (a) studies on adults (age ≥ 18 years) with CKD and T2DM; (b) the intervention should be nonsteroidal MRAs, compared with placebo; (c) trials with a duration longer than 12 weeks; (d) trials with at least one of the following outcomes: changes in urinary albumin to creatinine ratio (UACR), estimated glomerular filtration rate (eGFR), or systolic blood pressure (SBP) from baseline, incidence of hyperkalemia, eGFR decrease ≥30%, or total adverse events; (e) randomized controlled trial (RCT); (f) trials that reported at least one dispersion measure (confidence interval [CI], SD, or SE) for treatment groups.

Studies were excluded if any of the followings were identified: (a) animal studies, (b) nonoriginal analyses, (c) duplicate studies, (d) trials with nonplacebo control or short duration, (e) not an RCT, and (f) lack necessary data.

### 2.3 Data extraction and quality assessment

Data extraction was with standardized Excel forms by two independent reviewers, and disagreement was resolved by adjudicator reliance on the method, calibration, and traceability. Authors of articles were contacted for supplemental information when needed, and the references of selected articles were manually searched for additional relevant articles. The following data were extracted: research characteristics (first author, publication year, sample size, trial duration, treatment, and control), baseline patient characteristics (average age, sex ratio, proportion of Asian subjects, and conditions), and relevant outcomes. GetData Graph Digitizer software (version 2.2.5) was used to extract data presented as graphs. The primary end points were defined as renal outcomes, including the decrease in UACR and eGFR from baseline. The secondary end points consisted of SBP, hyperkalemia, eGFR decrease ≥30%, and total adverse events.

Two independent reviewers assessed the quality of each included study according to the Cochrane Handbook for Systematic Reviews of Interventions.^18^ The following factors were considered when evaluating the risk of bias: random sequence generation, allocation concealment, blinding of participants and investigators, blinding of outcome assessment, incomplete outcome data, selective outcome reporting, and other bias. The quality of evidence was assessed by using the Grading of Recommendations Assessment, Development and Evaluation (GRADE) profiler (GRADE pro) software (version 3.6.1). Details were placed in the Supporting Information (Figures [Link], [Link]).

### 2.4 Data synthesis and analysis

Review Manager (version 5.3) was used to perform statistical analysis. Except for 95% confidence interval (CI), we used weighted mean difference (WMD) for continuous outcomes and risk ratio (RR) for dichotomous outcomes. Statistical heterogeneity was assessed using the I^2^ test and random effects model. Sensitivity analyses were performed to detect the stability of the comparison results.

## 3 RESULTS

### 3.1 Eligible studies and study characteristics

**FIGURE 1:**
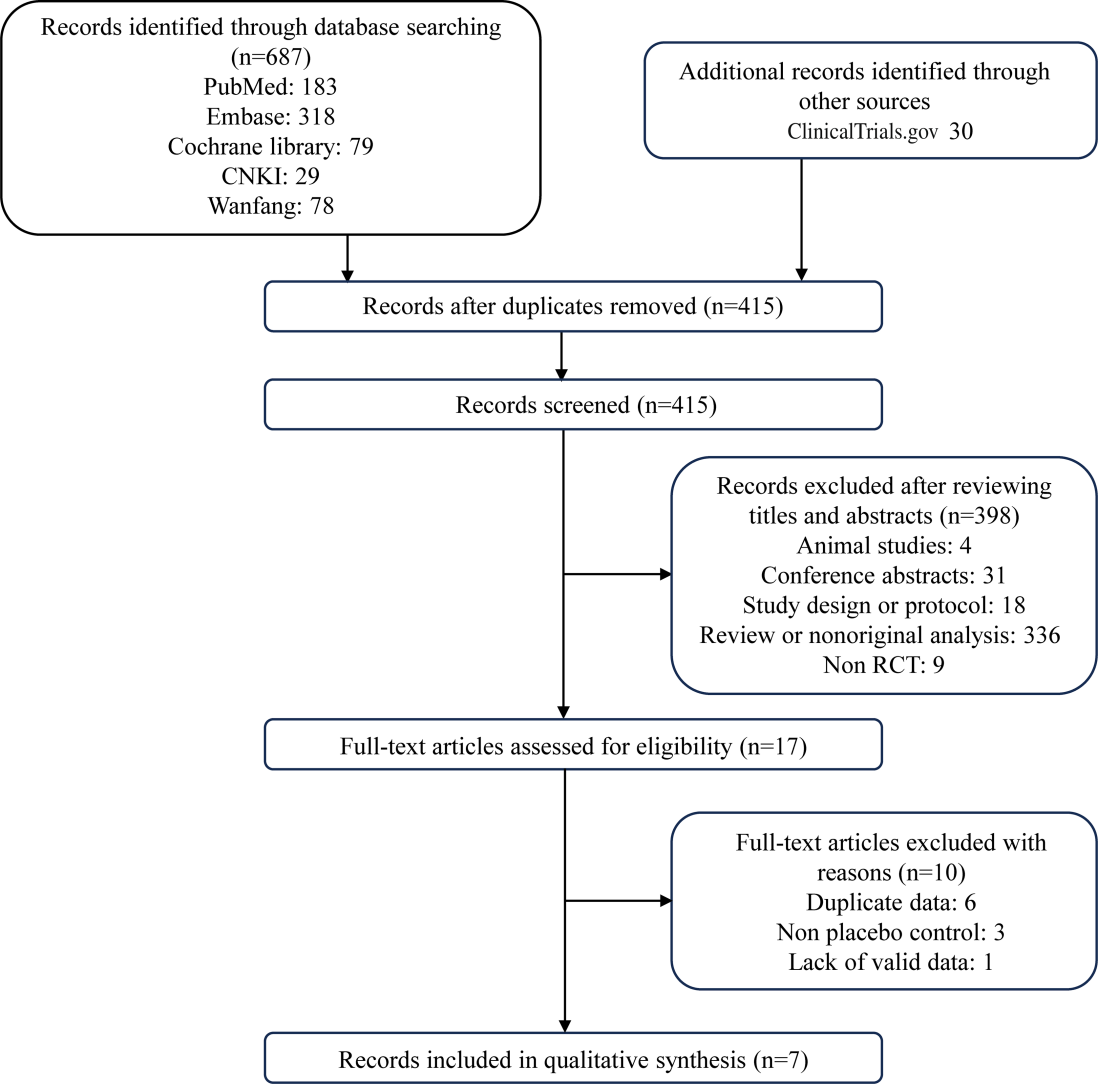
Flow diagram of study selection. CNKI, China National Knowledge Infrastructure; RCT, randomized controlled trial.

As shown in Figure 1, of the 717 studies (687 + 30) retrieved from databases and other sources, seven RCTs finally enrolled with 14 997 participants that met the inclusion criteria. For Asian subjects, there were three RCTs (843 patients).^19^, ^20^, ^21^ For non‐Asian subjects, four RCTs (14 154 patients) were included.^10^, ^11^, ^22^, ^23^ Nonsteroidal MRAs were given to experimental groups and control groups received placebo. The detailed baseline characteristics were summarized in Table 1.

**TABLE 1:** Characteristics of studies reporting the effects of nonsteroidal mineralocorticoid receptor antagonists (MRAs).

Source	Patients	Sample size, N	Treatment	Control	Duration	Study design	Age, years	Sex, male, n (%)	Race, Asian, n (%)	Hypertension n (%)	eGFR ≤ 60, mL/min/1.73 m^2^, n (%)	UACR ≥300, mg/g, n (%)	eGFR, mL/min/1.73 m^2^	UACR, mg/g, median (IQR)	Serum potassium, mmol/L	SBP, mm Hg	DBP, mm Hg	BMI, kg/m^2^
Bakris et al 2015	T2DM and persistent albuminuria	821	Finerenone	Placebo	12 weeks	Phase II, RCT	64.6 ± 9.2	639 (77.8)	84 (10.2)	774 (94.3)	328 (40.0)	301 (36.7)	67.5 ± 21.8	NA	4.3 ± 0.4	138.2 ± 14.4	77.1 ± 9.8	31.8 ± 5.5
Bakris et al 2020	CKD and T2DM	5734	Finerenone	Placebo	2.6 years	Phase III, RCT	65.6 ± 9.1	3983 (70.2)	1440 (25.4)	5505 (97.0)	5016 (88.4)	4963 (87.5)	44.3 ± 12.6	852 (446–1634)	4.4 ± 0.5	138.0 ± 14.4	75.8 ± 9.7	31.1 ± 6.0
Bakris et al 2021	Hypertension and Stage 3b/4 CKD and/or diabetes	162	Ocedurenone (KBP‐5074)	Placebo	22 weeks	Phase II, RCT	65.4 ± 11.5	89 (54.9)	0 (0)	162 (100.0)	162 (100)	85 (52.5)	31.9 ± 9.9	718	4.4 ± 0.4	155.3 ± 13.6	87.7 ± 12.2	NA
Pitt et al 2021	CKD and T2DM	7437	Finerenone	Placebo	3.4 years	Phase III, RCT	64.1 ± 9.8	5105 (69.4)	1454 (19.8)	7061 (96.0)	2812 (38.2)	3729 (50.7)	67.8 ± 21.7	308 (108–740)	4.3 ± 0.4	135.8 ± 14.0	76.8 ± 9.6	31.4 ± 6.0
Katayama et al 2017	T2DM and persistent albuminuria	96	Finerenone	Placebo	12 weeks	Phase II, RCT	63.0 ± 9.8	77 (80.2)	96 (100)	92 (95.8)	40 (41.7)	45 (46.9)	64.7 ± 14.1	249.2 ± 383.7	4.2 ± 0.4	138.7 ± 15.2	76.9 ± 11.2	27.0 ± 4.2
Ito et al 2020	Hypertension and T2DM with microalbuminuria	455	Esaxerenone (CS‐3150)	Placebo	52 weeks	Phase III, RCT	66 ± 9.0	345 (75.8)	455 (100)	455 (100.0)	145 (31.9)	0 (0)	69 ± 18.0	111 ± 46.3	4.4 ± 0.3	140 ± 10.0	84 ± 8.0	26.1 ± 4.0
Wada et al 2021	Stage 2 diabetic nephropathy	292	Apararenone (MT‐3995)	Placebo	24 weeks	Phase II, RCT	61.8 ± 9.3	221 (75.7)	292 (100)	NA	NA	0 (0)	74.9 ± 20.3	138.7 ± 83.5	4.3 ± 0.3	135.3 ± 11.9	77.9 ± 9.7	26.8 ± 4.6

*Note*: Values are reported as n (%), mean ± SD, or median (interquartile range) unless otherwise indicated.

Abbreviations: BMI, body mass index; CKD, chronic kidney disease; DBP, diastolic blood pressure; eGFR, estimated glomerular filtration rate; IQR, interquartile range; NA, not available; RCT, randomized controlled trial; SBP, systolic blood pressure; T2DM, type 2 diabetes mellitus; UACR, urinary albumin to creatinine ratio.

**FIGURE 2:**
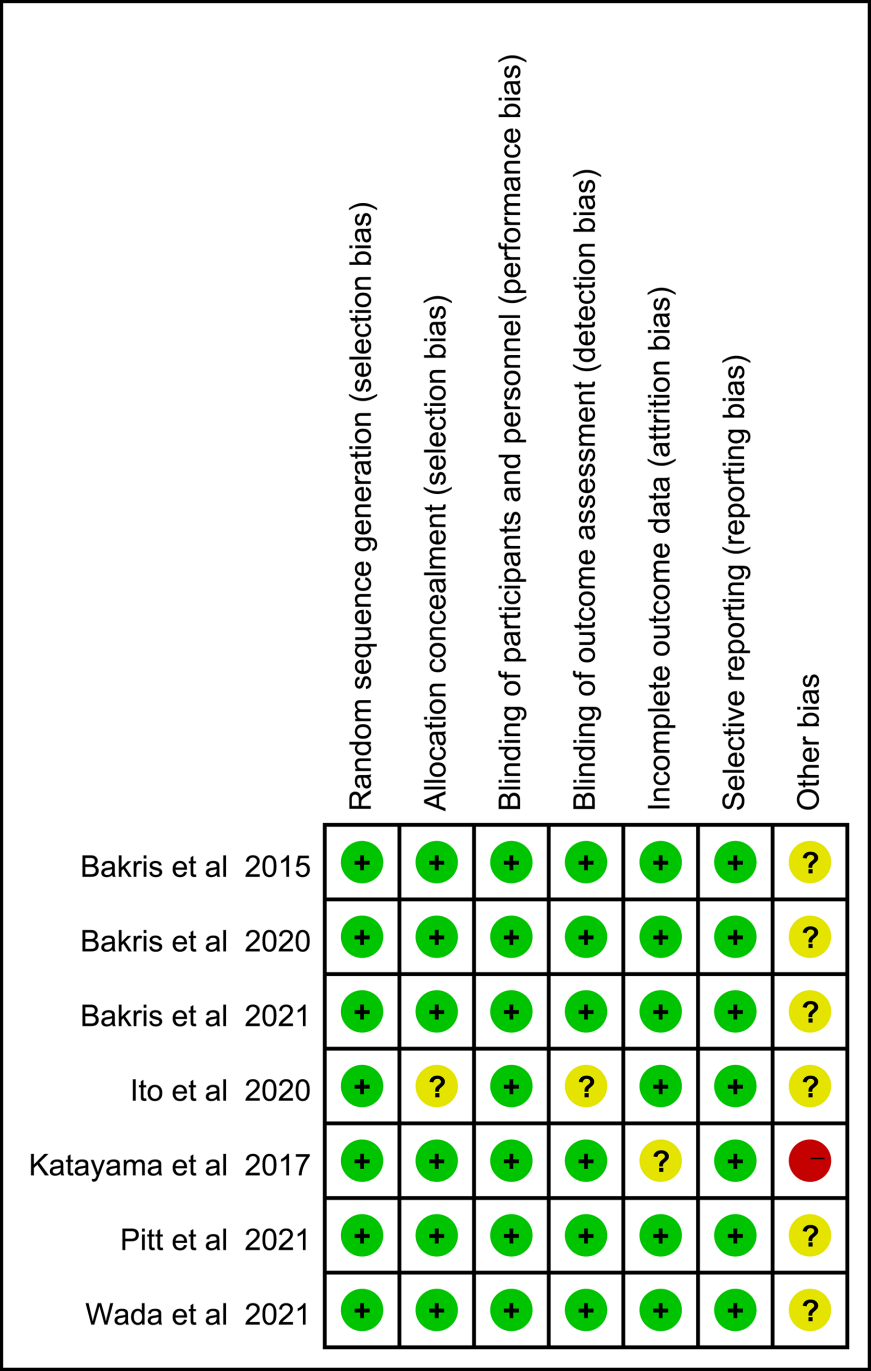
Evaluation of the risk of bias.

Among the included trials, one study contained a small sample with only 12 patients in control group, leading to “high risk” for weak evidence. The overall risk of bias was low, and details were presented in Figure 2.

### 3.2 Primary outcomes

**FIGURE 3:**
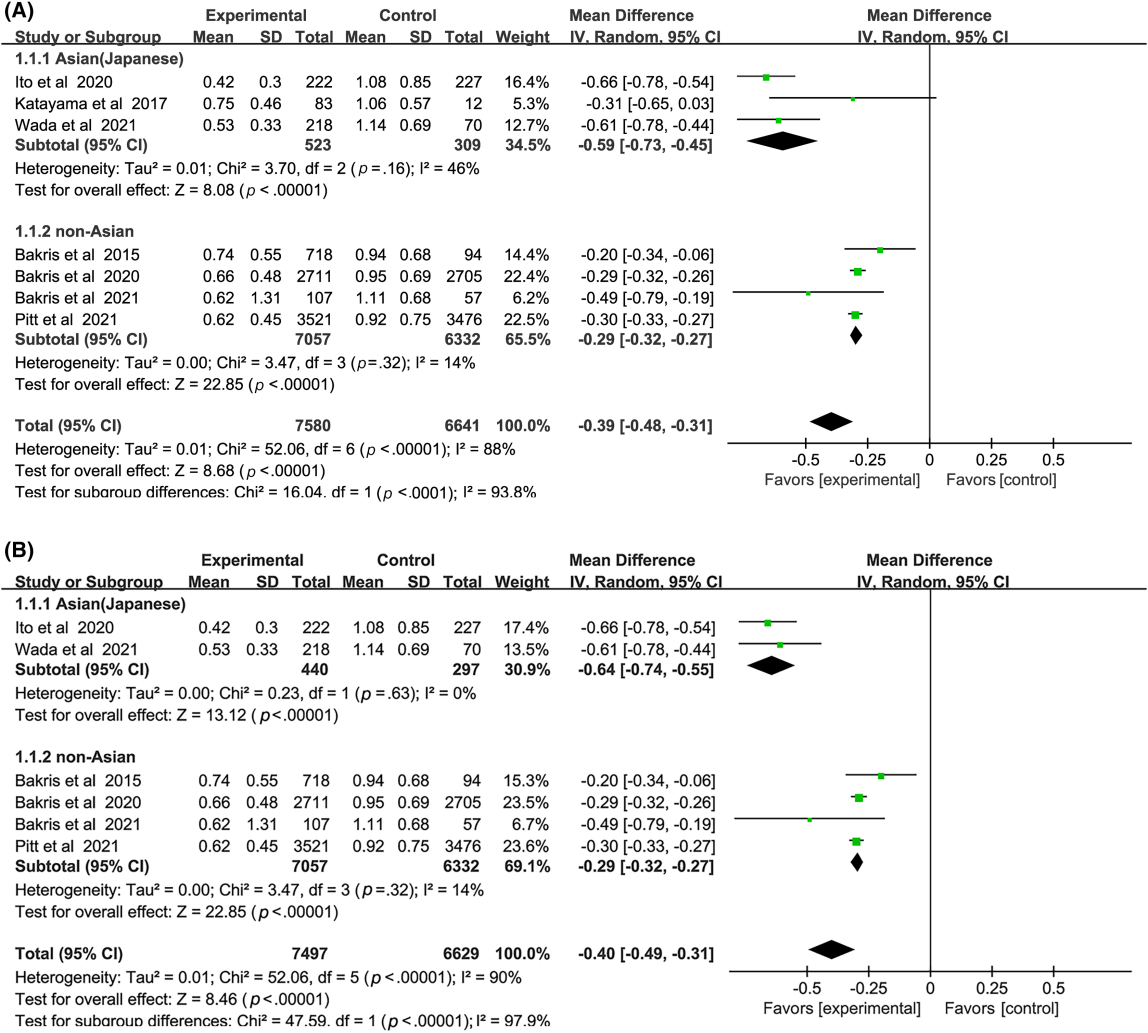
(A) Forest plot of the weighted mean difference in the change of urinary albumin to creatinine ratio (UACR) from baseline. (B) Forest plot of the weighted mean difference in the change of urinary albumin to creatinine ratio (UACR) from baseline. (Sensitivity analysis). CI, confidence interval; IV, interval variable.

Nonsteroidal MRAs reduced UACR in both Asian and non‐Asian patients compared with placebo (Figure 3A), and the decrease was significantly greater in the Asians (WMD, −0.59, 95% CI, −0.73 to −0.45, *p* < .01) than the non‐Asians (WMD, −0.29, 95% CI, −0.32 to −0.27, *p* < .01). The total heterogeneity was I^2^ = 88%, but the heterogeneities in subgroups were I^2^ = 46% and I^2^ = 14% respectively, indicating that the grouping factor was a significant source of heterogeneity.

Sensitivity analysis was performed by removing one study at a time to explore whether the heterogeneity changed (Figure 3B). The results did not alter: (WMD, −0.64, 95% CI, −0.74 to −0.55, *p* < .01) vs (WMD, −0.29, 95% CI, −0.32 to −0.27, *p* < .01) without heterogeneity in each subgroup. Test for subgroup differences: I^2^ = 97.9% (*p* < .01).

**FIGURE 4:**
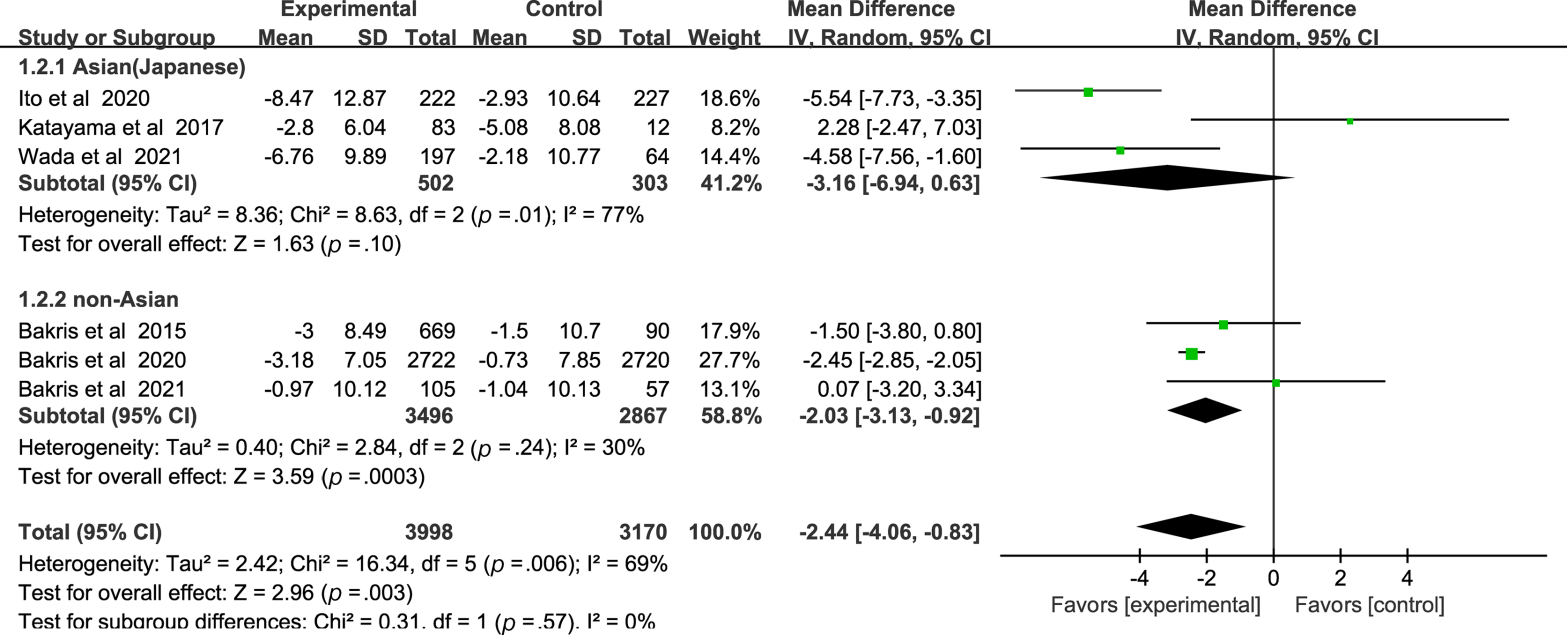
Forest plot of the weighted mean difference in the change of estimated glomerular filtration rate (eGFR) from baseline. CI, confidence interval; IV, interval variable.

As for eGFR results of nonsteroidal MRAs, there was no significant difference between Asian patients and non‐Asian patients: (WMD, −3.16, 95% CI, −6.94 to 0.63, *p* > .05) vs (WMD, −2.03, 95% CI, −3.13 to −0.92, *p* < .01), I^2^ = 77% and I^2^ = 30% respectively (Figure 4). Test for subgroup differences: I^2^ = 0% (*p* > .05).

### 3.3 Secondary outcomes

**FIGURE 5:**
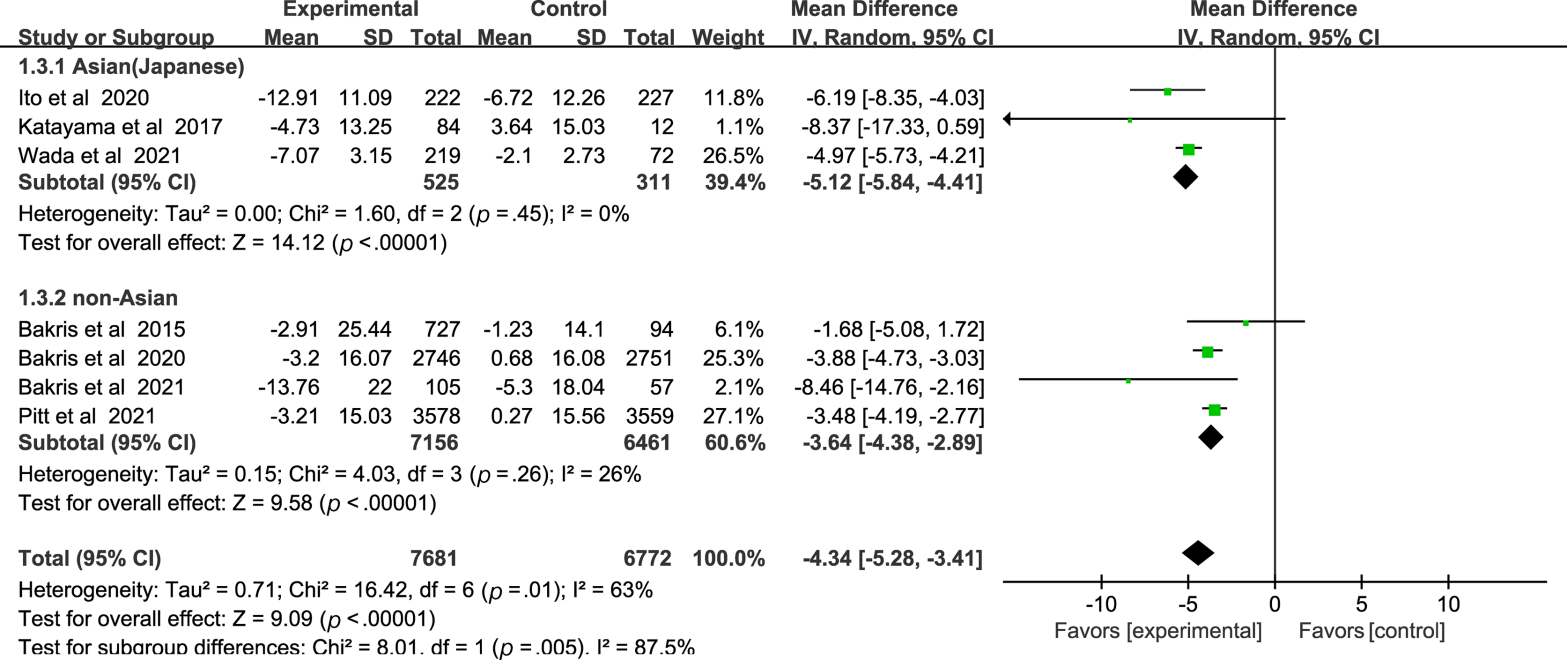
Forest plot of the weighted mean difference in the change of systolic blood pressure (SBP) from baseline. CI, confidence interval; IV, interval variable.

Compared with the placebo group, the nonsteroidal MRAs remarkably reduced SBP in patients with CKD and T2DM (WMD −4.34, 95% CI −5.28 to −3.41, *p* < .01), and more significantly in Asian patients than non‐Asian patients (WMD −5.12, 95% CI −5.84 to −4.41, *p* < .01) vs (WMD, −3.64; 95% CI, −4.38 to −2.89, *p* < .01), I^2^ = 0% and 26% respectively (Figure 5). Test for subgroup differences: I^2^ = 85.7% (*p* < .01).

**FIGURE 6:**
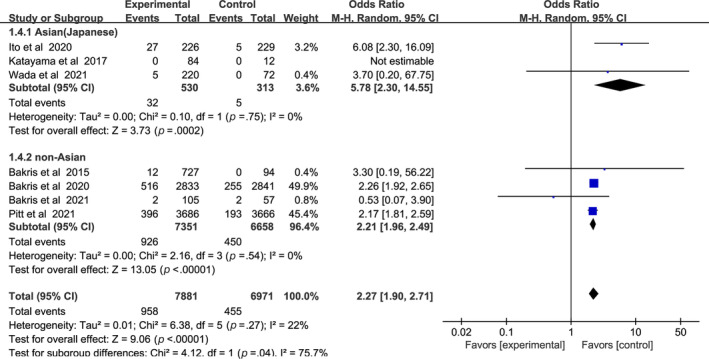
Forest plot of the odds ratio in the incidence of hyperkalemia. CI, confidence interval; M‐H, Mantel‐Haenszel.

In Figure 6, a higher incidence of hyperkalemia among patients with CKD and T2DM was found in the nonsteroidal MRA group vs placebo group (odds ratio [OR] 2.27, 95% CI 1.90–2.71, *p* < .01). There was no significant statistical heterogeneity (I^2^ = 22%) in the pooled effect estimate. Subgroup analysis showed that nonsteroidal MRAs in Asian patients presented a higher risk of hyperkalemia (OR 5.78, 95% CI 2.30–14.55, *p* < .01), compared with non‐Asian patients (OR 2.21, 95% CI 1.96–2.49, *p* < .01). Test for subgroup differences: I^2^ = 75.7% (*p* < .05).

**FIGURE 7:**
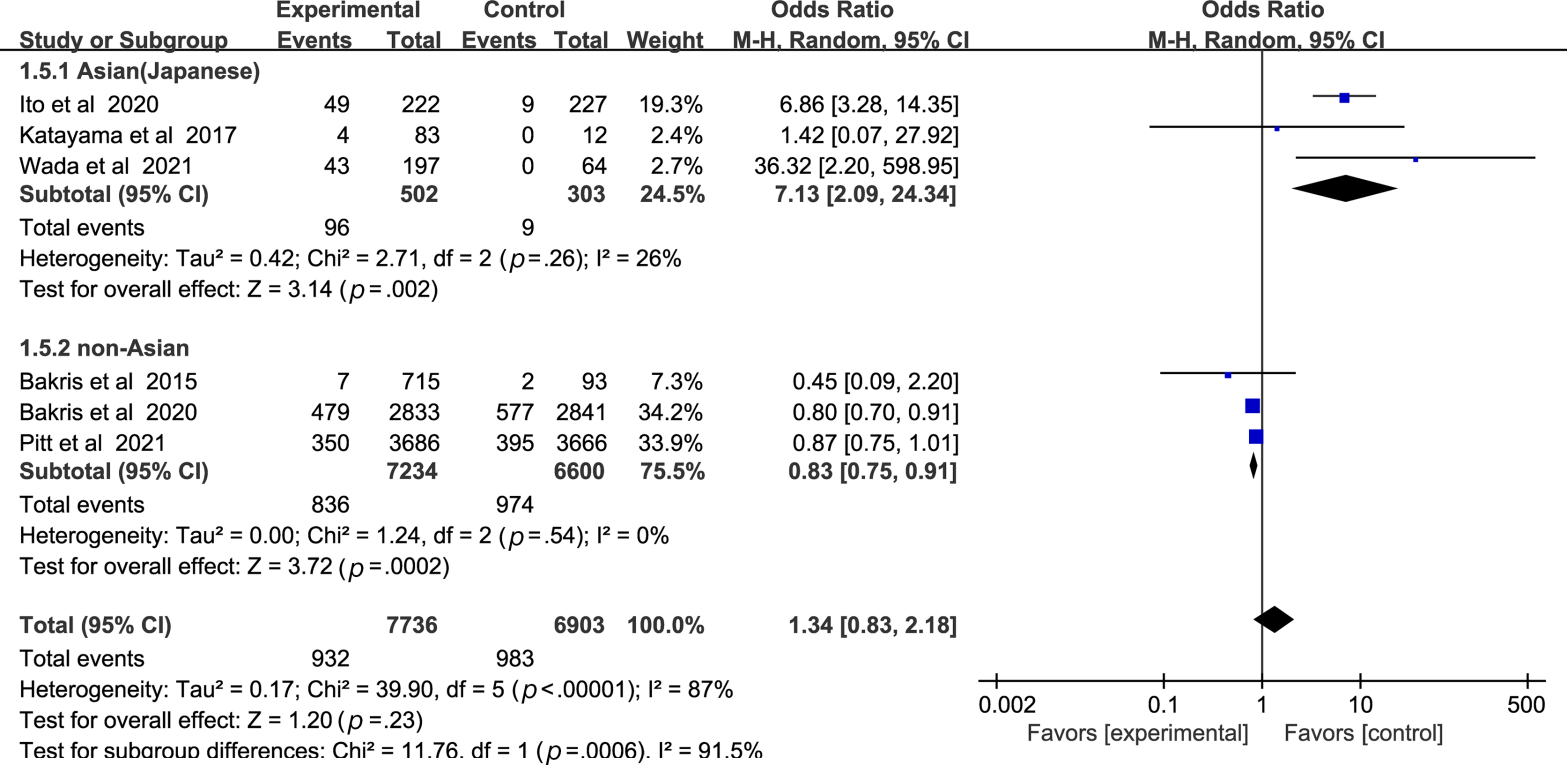
Forest plot of the odds ratio in the incidence of estimated glomerular filtration rate (eGFR) decrease ≥30%. CI, confidence interval; M‐H, Mantel‐Haenszel.

In Figure 7, the incidence of eGFR decrease ≥30% between nonsteroidal MRA group and placebo group was similar (OR 1.34, 95% CI 0.83–2.18, *p* > .05). Asian patients with nonsteroidal MRA were more likely to suffer sharp decrease of eGFR (OR 7.13, 95% CI 2.09–24.34, *p* < .01), whereas non‐Asian patients were opposite (OR 0.83, 95% CI 0.75–0.91, *p* < .01). Test for subgroup differences: I^2^ = 91.5% (*p* < .01).

**FIGURE 8:**
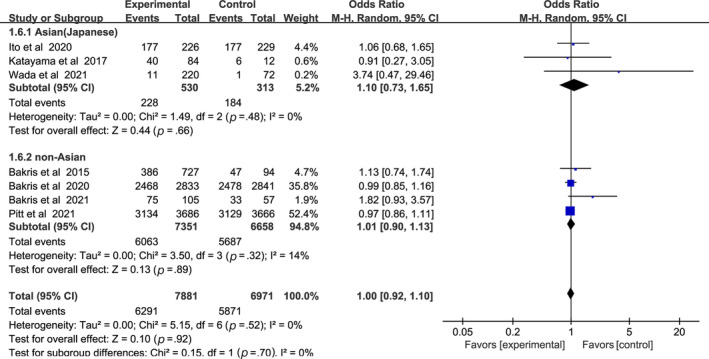
Forest plot of the odds ratio in the incidence of total adverse events. CI, confidence interval; M‐H, Mantel‐Haenszel.

In Figure 8, nonsteroidal MRAs did not show significant difference in total adverse events compared with placebo (OR 1.00, 95% CI 0.92–1.10, *p* > .05). The total heterogeneity among all trials was not significant (I^2^ = 0%). Test for subgroup differences: I^2^ = 0 (*p* > .05).

## 4 DISCUSSION

Diabetic nephropathy is the leading cause of chronic and end‐stage kidney disease.^24^ Up to 40% of patients with diabetes are expected to also develop CKD.^25^ Previous study indicated that the relationship between lowered eGFR and elevated plasma aldosterone might associated with overactivation of MR.^26^ Long‐term blockade of the renin–angiotensin system contributed to “aldosterone escape” or “aldosterone breakthrough,” a phenomenon that aldosterone levels increased in a subset of patients despite therapy.^27^, ^28^ Therefore, CKD patients still face a high residual risk.^29^ MRAs can block both aldosterone‐dependent and aldosterone‐independent MR signaling pathways, bringing direct anti‐inflammatory and antifibrosis effects.^30^, ^31^ Nonsteroidal MRAs have emerged as a new therapeutic tool targeting systemic impact.^32^, ^33^ Compared with steroidal MRAs, nonsteroidal MRAs show higher selectivity, affinity and more balanced tissue distribution between heart and kidney.^34^, ^35^ The finding about spironolactone generated the hypothesis that safety and efficacy of MRAs might differ by race.^36^ Thus, we conducted a meta‐analysis to evaluate the different renal outcomes between Asian and non‐Asian CKD and T2DM patients treated with nonsteroidal MRAs.

Our results indicated that nonsteroidal MRAs significantly decreased UACR in Asian patients compared with non‐Asian patients. The mean level of eGFR did not show obvious difference. As for SBP, nonsteroidal MRAs had a better antihypertension performance in Asians. Regarding safety, the incidence of total adverse events did not show significant differences compared with placebo, neither for Asians nor non‐Asians. Hyperkalemia and eGFR decrease ≥30% occurred more frequently in Asian patients treated with nonsteroidal MRAs. Although the incidence of serum potassium >5.5 mmol/L was higher than that in the non‐Asian subgroup, there was no death due to hyperkalemia. Notably, even a 30% reduction in eGFR was acceptable depending on the magnitude of the decrease in SBP, and these patients did not present adverse reactions associated with renal function.^37^ As for hyperkalemia, it is suggested that concomitant use of sodium‐glucose cotransporter 2 inhibitor can reduce the risk in Japanese patients treated with esaxerenone.^38^ Doctors should adjust dosage of nonsteroidal MRAs or add other medications under comprehensive consideration. Close monitoring of SBP, K^+^, and eGFR does matter in Asian patients.

The discrepancy may be explained by the following reasons. First, the dietary habit of Asians tends to high sodium food. Indeed, the dietary factors such as high salt, sugar, and fat, alone or in various permutations, are supposed to promote the onset and development of cardiovascular, renal, and endocrine disorders.^39^, ^40^ Excessive salt intake triggers renal Rac1 upregulation, followed by increased Sgk1 expressions, a downstream molecule of MR signal, indicating salt‐induced activation of Rac1‐MR pathway.^41^, ^42^ In kidney, MR activation promotes the transcription of the basolateral Na^+^/K^+^‐ATPase and the apical epithelial Na^+^ channel, resulting in the increasing reabsorption of Na^+^ and excretion of K^+^.^30^, ^43^, ^44^ Owing to MR blockade, patients receiving MRAs have lower blood pressure and a higher incidence of hyperkalemia.^45^ Second, there are differences in population allele frequences, leading to MR polymorphism by race. For example, variants in NR3C2 (nuclear receptor superfamily 3, group C, member 2) genotype, which codes the target protein MR, are associated with cardiorenal response to spironolactone.^46^, ^47^ Besides, Asians possess lower body weight than non‐Asians.^48^ Therefore, the drug doses (amount of drug/kg) of nonsteroidal MRAs are relatively higher in Asian patients.

There are limitations in our study. First, the non‐Asian subgroup enrolled multicenter studies that did not eliminate a small proportion of Asians. Although it was not completely comparable, the significance of races had been proved without other predominant variables. Second, only seven studies with 14 997 subjects were included. Trials from other Asian countries except Japan were not found in the databases, making the Asian participants less representative. Subsequent research should strive to encompass a more diverse demographic within the Asian population. Third, the administration duration of nonsteroidal MRAs varied from 90 days to 3.4 years may cause bias.^49^ Given these limitations, our results should be interpreted with caution.

## 5 CONCLUSIONS

Our study indicated that nonsteroidal MRAs decreased UACR and SBP significantly greater in Asian CKD and T2DM patients than non‐Asian patients. The average decline in eGFR did not show racial preference.

There was no significant difference in total adverse events. However, hyperkalemia and eGFR decrease ≥30% occurred more frequently in Asians, which was acceptable without renal failure or death. Doctors should pay more attention to these early changes in patients taking nonsteroidal MRAs, especially in Asians. In the future, we anticipate research of nonsteroidal MRAs with a particular emphasis on diverse populations and investigating long‐term outcomes. Participants can be categorized into various groups, including different doses, sex, and different ranks of proteinuria and eGFR, to enhance the individual management of renal disease.

## AUTHOR CONTRIBUTIONS

Xiaoming Xu conceived the meta‐analysis and contributed to the planning, methodology, drafting, and revision of the manuscript. Jing Feng and Yuying Cui performed systematic searches, study selection, and data extraction and analysis. Xiaoming Xu and Pingjiang Li wrote the first draft, with adjudications made by Lin Liao and Jianjun Dong. All authors contributed to the writing and revision of the manuscript. All authors approved the final draft of the manuscript.

## CONFLICT OF INTEREST STATEMENT

The authors declare that the research was conducted in the absence of any commercial or financial relationships that could be construed as a potential conflict of interest.

## Supporting information


**Figure S1.** GRADE approach to assess the overall confidence for urinary albumin to creatinine ratio (UACR). GRADE, Grading of Recommendations Assessment, Development and Evaluation; MRA, mineralocorticoid antagonist.

**Figure S2.** GRADE approach to assess the overall confidence for estimated glomerular filtration rate (eGFR). GRADE, Grading of Recommendations Assessment, Development and Evaluation; MRA, mineralocorticoid antagonist.

**Figure S3.** GRADE approach to assess the overall confidence for systolic blood pressure (SBP). GRADE, Grading of Recommendations Assessment, Development and Evaluation; MRA, mineralocorticoid antagonist.

**Figure S4.** GRADE approach to assess the overall confidence for hyperkalemia. GRADE, Grading of Recommendations Assessment, Development and Evaluation; MRA, mineralocorticoid antagonist.

**Figure S5.** GRADE approach to assess the overall confidence for estimated glomerular filtration rate (eGFR) decrease ≥ 30%. GRADE, Grading of Recommendations Assessment, Development and Evaluation; MRA, mineralocorticoid antagonist.

**Figure S6.** GRADE approach to assess the overall confidence for total adverse events. GRADE, Grading of Recommendations Assessment, Development and Evaluation; MRA, mineralocorticoid antagonist.
