# Supplementary figures and images for: Renal effects and safety between Asian and non‐Asian chronic kidney disease and type 2 diabetes treated with nonsteroidal mineralocorticoid antagonists
Source: J Diabetes. 2024 May 16;16(6):e13566. doi: 10.1111/1753-0407.13566 (PMC11098447; doi:10.1111/1753-0407.13566)

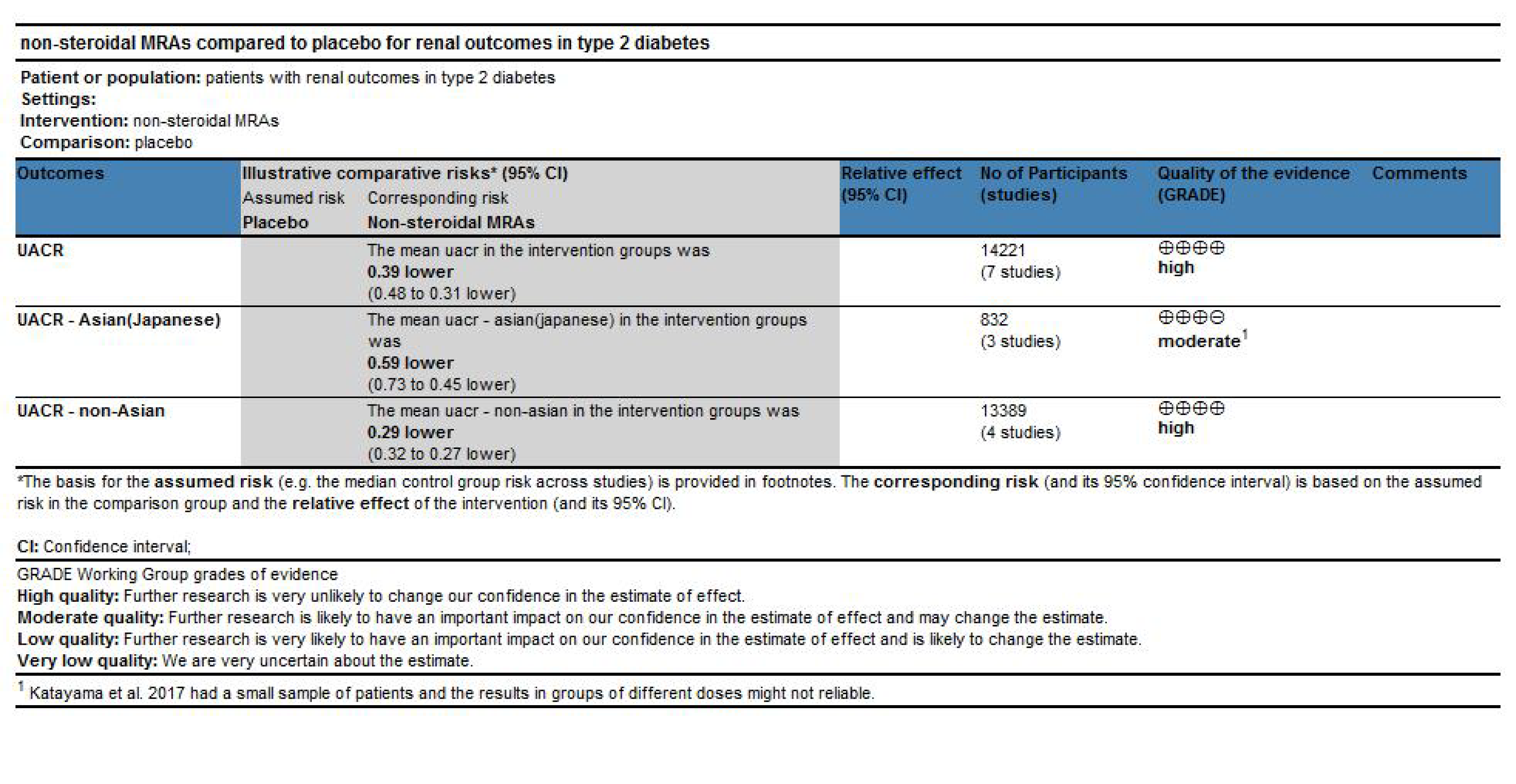

Supplement: Supplementary file 1 — Figure S1. GRADE approach to assess the overall confidence for urinary albumin to creatinine ratio (UACR). GRADE, Grading of Recommendations Assessment, Development and Evaluation; MRA, mineralocorticoid antagonist. [file JDB-16-e13566-s002.tif]

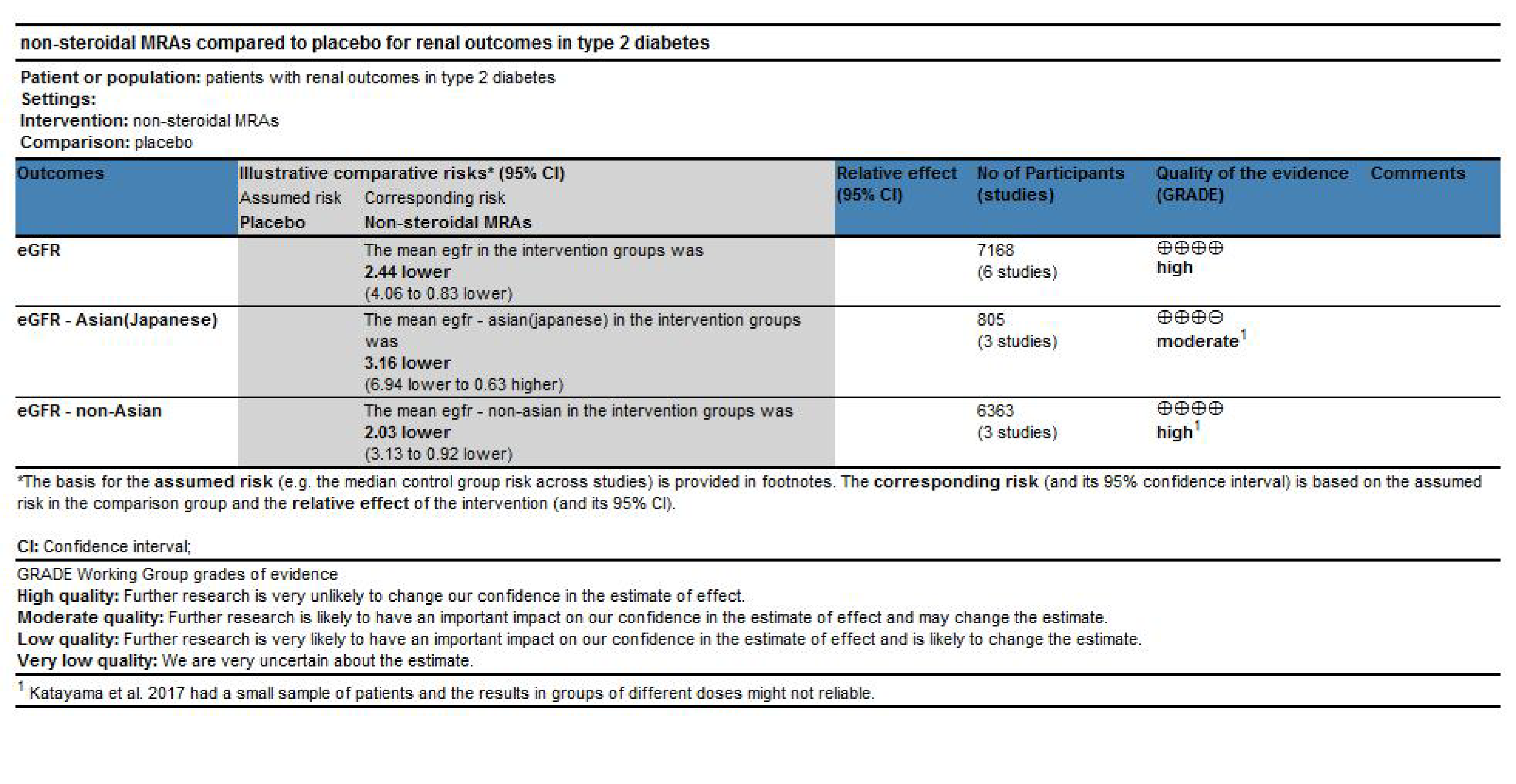

Supplement: Supplementary file 2 — Figure S2. GRADE approach to assess the overall confidence for estimated glomerular filtration rate (eGFR). GRADE, Grading of Recommendations Assessment, Development and Evaluation; MRA, mineralocorticoid antagonist. [file JDB-16-e13566-s003.tif]

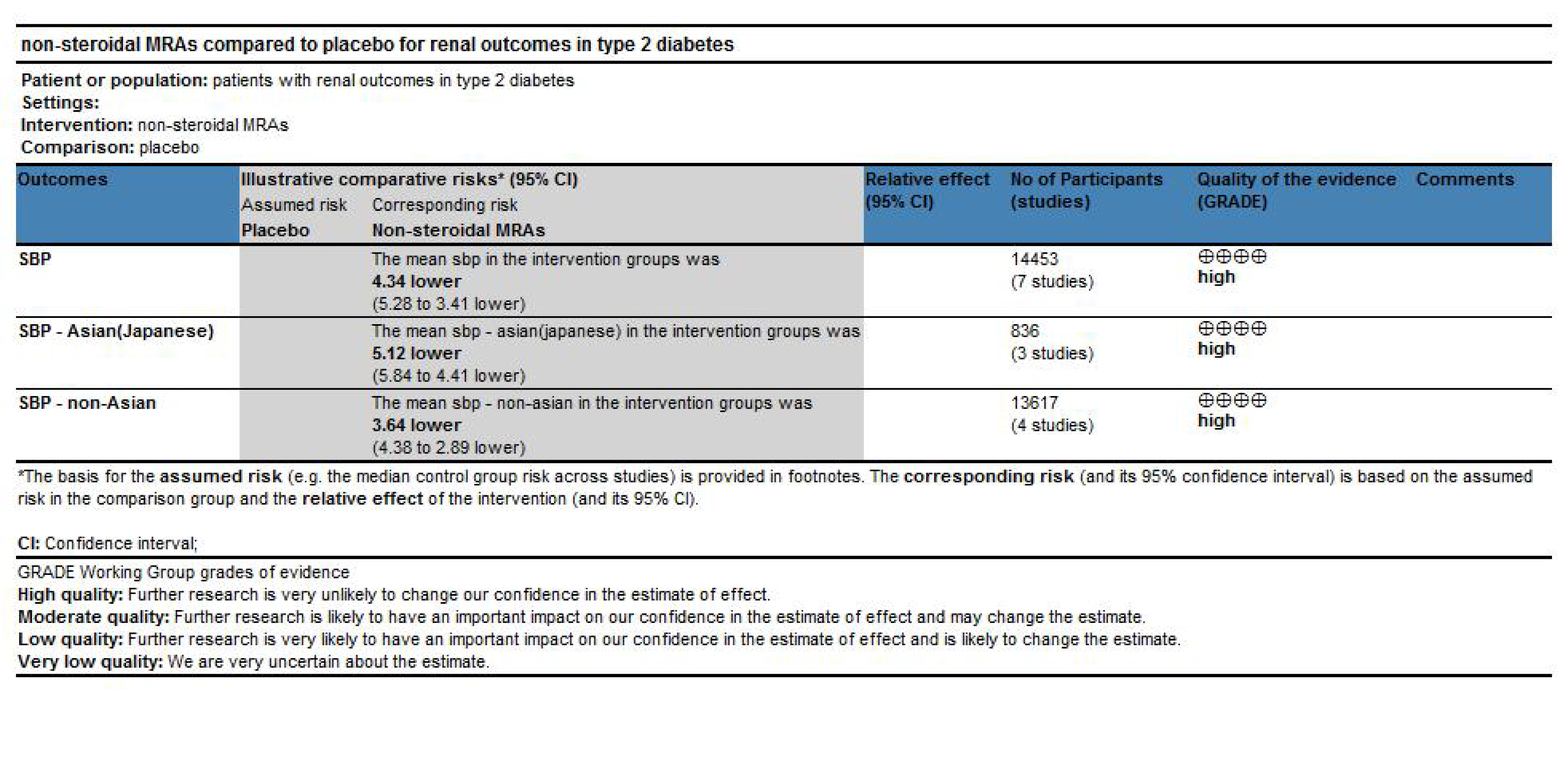

Supplement: Supplementary file 3 — Figure S3. GRADE approach to assess the overall confidence for systolic blood pressure (SBP). GRADE, Grading of Recommendations Assessment, Development and Evaluation; MRA, mineralocorticoid antagonist. [file JDB-16-e13566-s004.tif]

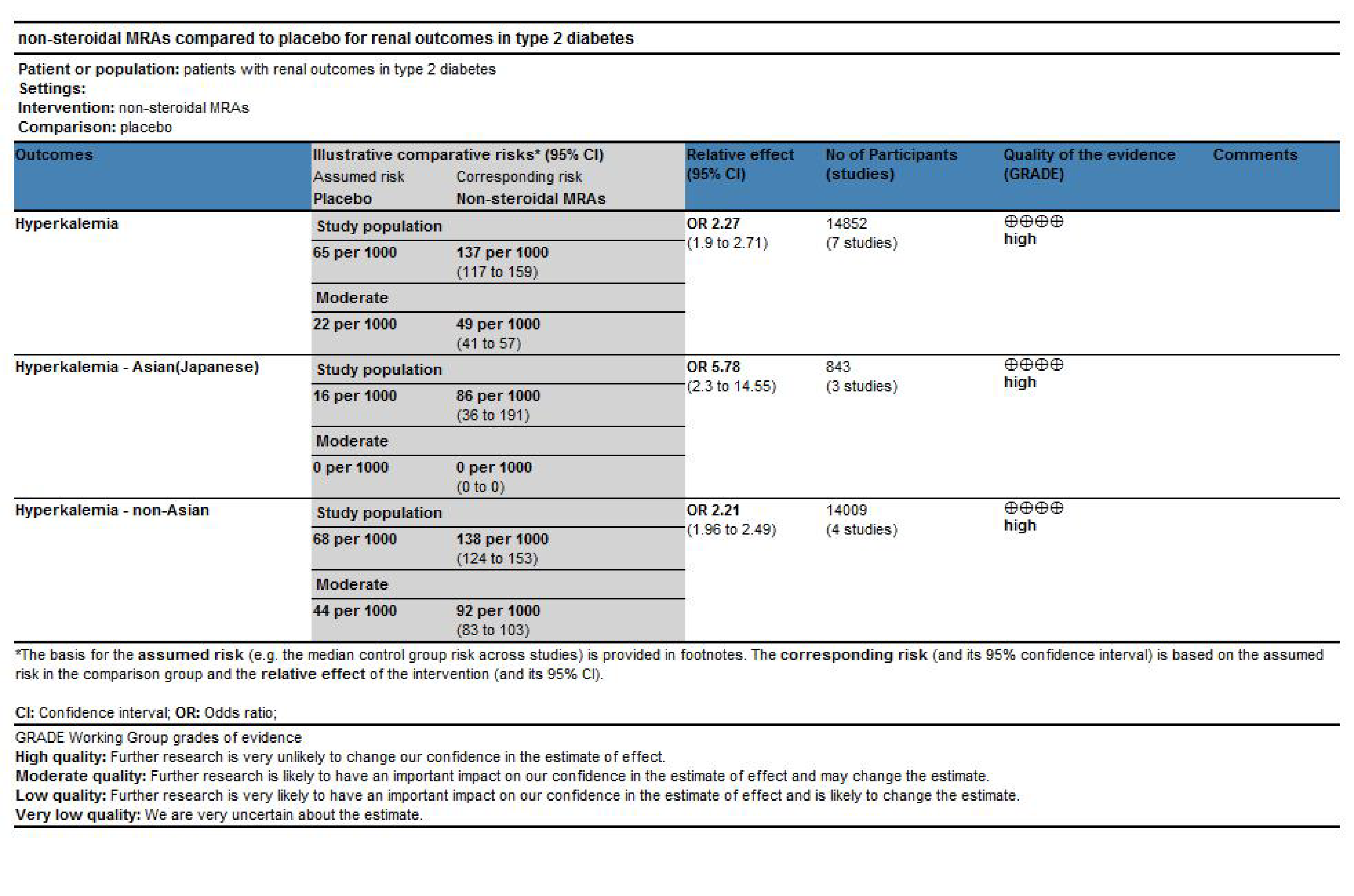

Supplement: Supplementary file 4 — Figure S4. GRADE approach to assess the overall confidence for hyperkalemia. GRADE, Grading of Recommendations Assessment, Development and Evaluation; MRA, mineralocorticoid antagonist. [file JDB-16-e13566-s001.tif]

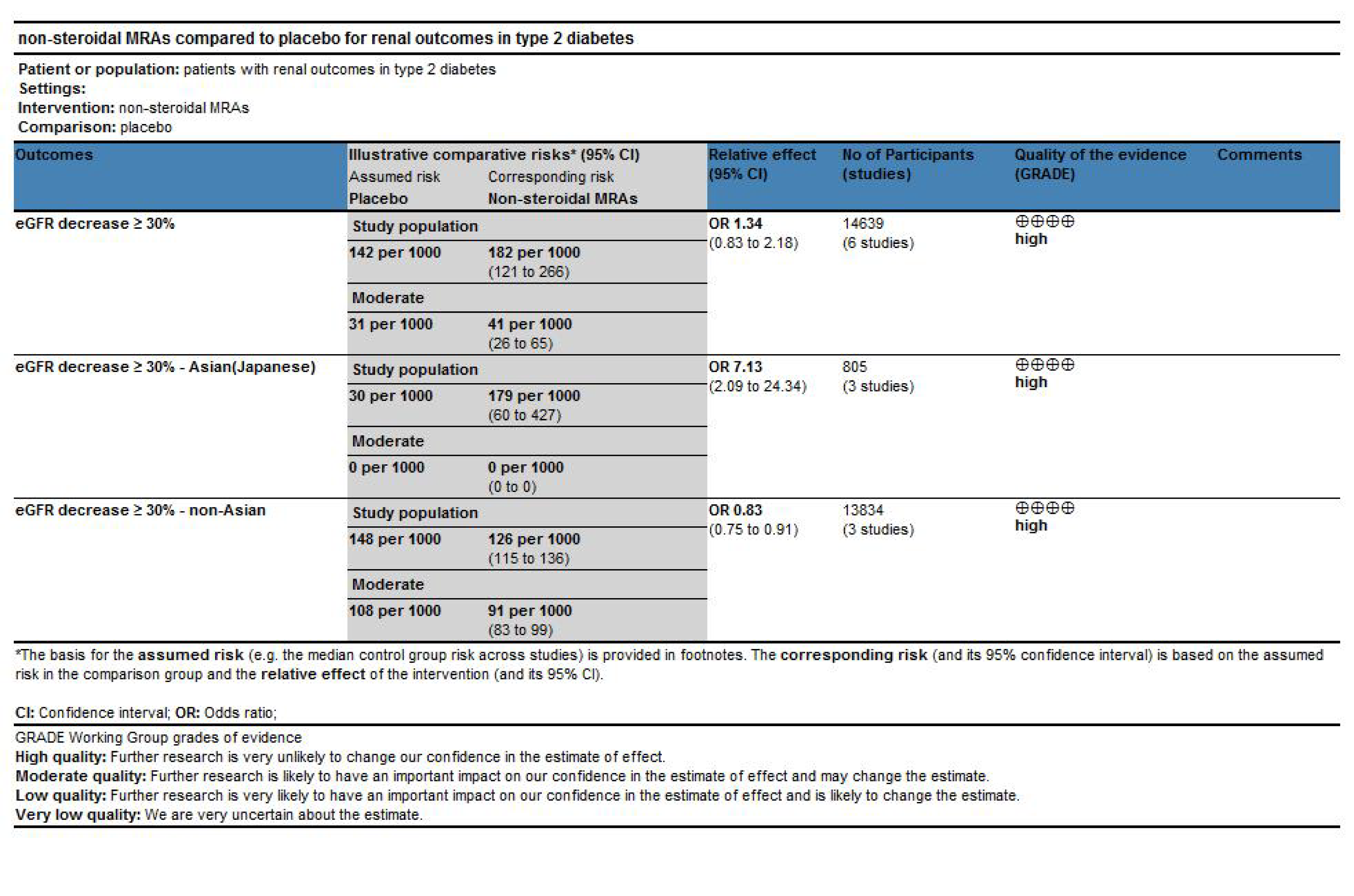

Supplement: Supplementary file 5 — Figure S5. GRADE approach to assess the overall confidence for estimated glomerular filtration rate (eGFR) decrease ≥ 30%. GRADE, Grading of Recommendations Assessment, Development and Evaluation; MRA, mineralocorticoid antagonist. [file JDB-16-e13566-s005.tif]

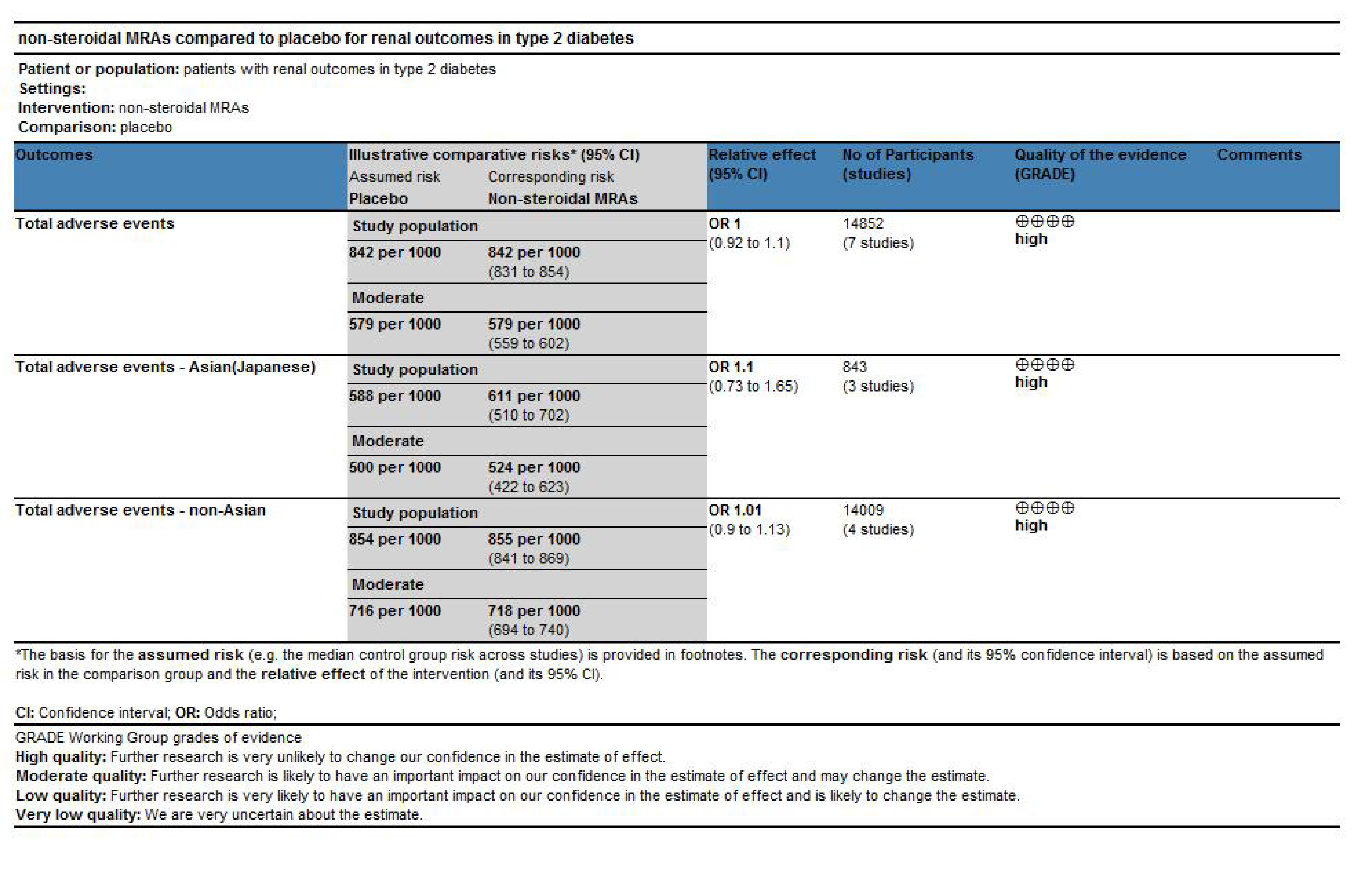

Supplement: Supplementary file 6 — Figure S6. GRADE approach to assess the overall confidence for total adverse events. GRADE, Grading of Recommendations Assessment, Development and Evaluation; MRA, mineralocorticoid antagonist. [file JDB-16-e13566-s006.tif]
